# A study on the sports behavior and motivation of young baseball players

**DOI:** 10.3389/fpubh.2026.1734154

**Published:** 2026-04-07

**Authors:** Chao-Fu Chen, Lin Zhu, Teng-Xiang Ge, He-Qian Liu, Ya-Zhen Cui, Hui-Ju Wu

**Affiliations:** 1 Physical Education College, Jimei University, Xiamen, China; 2 Physical Education College, Zhengzhou University of Industrial Technology, Zhengzhou, China; 3 Physical Education Department, Xiangtan University, Xiangtan, China; 4 Physical Education College, Huaibei Normal University, Huaibei, China; 5 China Football College, Beijing Sport University, Beijing, China; 6 Xiamen Yixue Sports and Technology Co., Ltd., Xiamen, China

**Keywords:** female, physical activity, regression analysis, sports, sports psychology

## Abstract

**Introduction:**

This study aims to investigate the relationship between participation motivation and exercise behavior among adolescent leisure baseball enthusiasts.

**Methods:**

A questionnaire survey was used to collect data, resulting in 919 valid questionnaires, including 484 males and 435 females.

**Results:**

Results revealed significant gender differences in psychological motivation, enjoyment motivation, and exercise behavior among recreational baseball enthusiasts. Males outperformed females on all indicators, indicating a higher level of engagement and willingness to participate in baseball. Correlation analysis revealed significant positive correlations between exercise behavior and life motivation, psychological motivation, and enjoyment motivation, indicating that higher motivation levels were associated with higher frequency and persistence in actual exercise participation. Further regression analysis revealed that exercise behavior was significantly influenced by life motivation and enjoyment motivation, suggesting that viewing exercise as an important part of life and deriving pleasure and a sense of accomplishment from it can contribute to baseball participation.

**Conclusion:**

Overall, these findings suggest that motivation plays a key role in recreational baseball participation, providing valuable insights for promotional activities and sports education.

## 1 Introduction

Physical activity plays an important role in promoting physical and mental health, as well as supporting social development and psychological adjustment. Baseball was selected as the focal sport because it integrates technical skill, teamwork, and long-term engagement, making it particularly suitable for examining motivational mechanisms in youth sport participation. Adolescence represents a critical period for the development of values and behavioral habits; therefore, understanding motivations for baseball participation may help promote sustained engagement and inform the design of effective physical education and training programs (1).

Motivation is a key psychological factor influencing individuals’ choices, effort, and persistence in sport. According to self-determination theory (SDT) (2), motivation can be broadly categorized into intrinsic and extrinsic forms. Intrinsic motivation reflects participation driven by enjoyment, interest, and personal satisfaction, whereas extrinsic motivation involves engagement influenced by external rewards, social recognition, or expectations. Both forms of motivation have been shown to affect adolescents’ commitment to sport participation (1).

Adolescents’ sport motivations are dynamic and shaped by both personal experiences and environmental factors. While some individuals gradually develop intrinsic motivation through skill mastery and enjoyment, others remain primarily influenced by external pressures such as parental expectations or peer recognition (3). An imbalance favoring external regulation may increase the risk of burnout and sport withdrawal, highlighting the importance of fostering autonomous and enjoyable sport experiences (2).

Social and cultural contexts further influence youth sport motivation. Factors such as family support, coaching style, school sport systems, and peer relationships can either facilitate or hinder sustained participation. Supportive coaching environments that emphasize autonomy and competence have been shown to enhance intrinsic motivation, whereas controlling approaches may reduce long-term engagement (3–5).

Therefore, a clearer understanding of motivational factors underlying youth baseball participation has practical implications for coaches, educators, and policymakers. By identifying motivational patterns associated with exercise behavior, this study aims to examine the relationships between sport participation motivation, demographic variables, and exercise behavior among youth baseball enthusiasts. The findings are intended to provide empirical evidence to support more effective and sustainable youth baseball promotion strategies.

## 2 Literature interview and build research hypotheses

### 2.1 Definition and types of motivation

Motivation is the psychological factor that drives individuals to work toward something or a goal that they find meaningful (6). It is a diverse and complex psychological state that can be divided into different types according to the source and nature of motivation, such as intrinsic motivation, extrinsic motivation, physiological motivation, psycho-logical motivation and social motivation; Intrinsic motivation: refers to individuals’ spontaneous participation in activities because they enjoy the challenge, happiness and sense of accomplishment brought by the process (7); extrinsic motivation: refers to individuals’ purposeful participation in activities, mainly to obtain material rewards or avoid punishment (8); physiological motivation: it is a biological driving force caused by insufficient physiological needs, such as hunger, thirst, etc. (9); psychological motivation: refers to the inner motivation of an individual, such as exploration, curiosity and expectation, etc. (10); social motivation: refers to the need for cooperation in interpersonal interactions. Individuals are satisfied by interacting with others, such as a sense of companionship, reciprocity, and a sense of belonging (11).

To sum up the above, motivation is the inner psychological factor that guides our actions, and it plays an extremely important role in our daily lives. Recognizing different kinds of motivation is critical to understanding one’s own and others’ behavior and decision-making processes. Motivation is what drives us to action, and different types of motivation are effective in different situations. Recognizing an individual’s motivational style can help us understand their behavior and choices while maintaining motivation and enthusiasm in the pursuit of goals and dreams.

### 2.2 Definition of exercise behavior

According to the American College of Sports Medicine (1990), to improve or maintain cardiorespiratory fitness, adults are recommended to perform aerobic exercise 3 to 5 times a week, lasting 20 to 60 min each time. Intensity should be 60 to 90 percent of your maximum heart rate or 50 to 80 percent of your VO_2_ max. Movement behavior can be understood from three aspects: type, frequency and intensity of movement, as follows (12):

Type of exercise: baseball; exercise frequency: the average number of times an individual exercises per week and the average duration of each exercise; exercise intensity: measure the intensity of exercise by measuring your heart rate or the Perceived Exercise Intensity Scale.

Past research results show that the type, intensity, frequency and duration of exercise had a positive impact on physical health and fitness. Appropriate exercise promotes health, improves physical mobility, and enriches quality of life (13). This has long been recognized as the value of sport. Establishing a consistent exercise routine is critical not only for physical development and health, but also for reducing stress and promoting human interaction (14). When targeting exercise specifically for adolescents, frequency, intensity, and duration should be considered. Research points out that extending the du-ration of low- to moderate-intensity exercise can produce effects similar to those of high-intensity exercise, while also contributing to physical and mental development. In addition, low-to-moderate intensity exercise is more acceptable, which means it is easier for teenagers to form exercise habits (15, 16). Overall, exercise not only has physical benefits, but also has a positive impact on mental health and social interactions. We should encourage young people to develop good exercise habits and develop appropriate exercise plans based on their needs and circumstances. This will contribute to the all-round development of young people while also promoting the health and happiness of society as a whole.

### 2.3 Research hypotheses

The purpose of this study was to understand the different background variables, sports participation motivations and sports behaviors of youth baseball enthusiasts. The research hypotheses were as follows:

*H1*: Youth baseball enthusiasts of different genders had significant differences in their motivations for sports participation.

*H2*: There were significant differences in exercise behavior among youth baseball enthusiasts of different genders.

*H3*: Sports participation motivation was significantly related to sports behavior.

*H4*: There was predictive power between sports participation motivation and sports behavior.

## 3 Materials and methods

### 3.1 Research participants

The participants were adolescents aged 14 to 18, recruited from schools and sports clubs in Mainland China.

### 3.2 Research methods

This study employed a non-probability convenience sampling method. The participants were adolescents recruited from schools and sports clubs in Mainland China. Data collection was conducted from June to September 2025. The inclusion criteria were as follows: (1) aged between 14 and 18 years, (2) participating in exercise at least twice a week, and (3) voluntary participation with a signed informed consent form. Exclusion criteria included: (1) questionnaires with more than 20% missing data, and (2) responses exhibiting obvious patterns of consistent or repetitive answering. The Academic Ethics Committee of Jimei University, with approval number: 20250609908. Written parental consent and participant assent were obtained prior to data collection.

The questionnaire method was used to collect data. The content of the questionnaire was divided into three parts:

The first part was the Physical Activity and Leisure Motivation Scale (PALMS), which refers to the items specially designed for youth baseball enthusiasts and adults by Molanorouzi et al. (17). It had eight subfacets (competition, appearance, life, social interaction, health, psychology, ability, fun), each facet consists of five questions, which can accurately reflect the motivation of the ethnic group to participate in sports. A five-point Likert scale was used, and the participants choose one of the five options to answer the statement based on the content of the scale and according to their personal motivation for exercise. The scoring method was: the answer “strongly agree” was 5 points, “agree” was 4 points, “no opinion” was 3 points, “disagree” was 2 points, and “strongly disagree” was 1 point. The higher the score, the higher the sports motivation score of this aspect.

The second part was the exercise behavior scale, which was adapted from Godin’s leisure time fitness exercise questionnaire, with a total of three questions (18). Respondents were asked to recall the frequency and duration of vigorous physical activity, moderate physical activity and light physical activity for at least 15 min in the past month, and then calculate the metabolic equivalent index corresponding to various physical activity intensity. The calculation formula was as follows: vigorous physical activity (9 points) * Number of exercise days + moderate physical activity (5 points) * Number of exercise days + light physical activity (3 points) * Number of exercise days = total score of exercise behavior. The higher the total score, the more exercise behavior. Otherwise it was low.

The third part was basic personal information.

The original English version of the scale underwent a forward and backward translation process by bilingual experts, and its semantic appropriateness was reviewed by three experts in sports psychology. Following a pilot test with 30 participants, the items were confirmed to be semantically clear, and no further revisions were made.

### 3.3 Data analysis process

The validity of the “Physical Activity and Leisure Motivation Scale” questionnaire used Cronbach’s alpha coefficient to test the internal consistency. Used descriptive statistics analysis to test the mean and standard deviation of sports motivation and behavior. Used independent samples t-test to test the significant differences in sports participation motivation and sports behavior among youth baseball enthusiasts of different genders. Pearson product–moment correlation analysis was used to test the relationship between the two variables of sports participation motivation and sports behavior. Stepwise multiple regression was employed as an exploratory approach to identify the most salient motivational predictors of exercise behavior. Although this method is data-driven, it was used here to complement theory-based analyses and to provide an initial empirical screening of relevant motivational dimensions.

## 4 Results

A total of 945 questionnaires were collected. After excluding 26 invalid responses, 919 valid questionnaires remained (484 males and 435 females). Prior to data analysis, normality was assessed using the Shapiro–Wilk test, alongside skewness and kurtosis values. The skewness and kurtosis for all variables fell within the range of ±2, satisfying the assumption of a normal distribution. Homogeneity of variance was examined using Levene’s test, and the results did not violate the assumption (*p* > 0.05). Before conducting multiple regression analysis, multicollinearity was examined; the variance inflation factor (VIF) for each independent variable was below 2, indicating no significant multicollinearity issues. 95% confidence interval. The internal consistency reliability for each dimension ranged from *α* = 0.89 to 0.98, demonstrating that the scale possesses excellent internal consistency (19) (Table 1).

**Table 1:** Reliability analysis.

Factor	Cronbach’s alpha	
Competition	0.945	0.984
Appearance	0.949	
Life	0.894	
Social interaction	0.960	
Healthy	0.985	
Psychology	0.966	
Ability	0.966	
Fun	0.975	

The exercise behavior scale shows that 58.87% of youth baseball enthusiasts engage in vigorous physical activity (an average of 3.39 days a week, about 39.56 min a day), 59.96% engage in moderate physical activity (an average of 3.26 days a week, about 40.96 min a day), and 64.53% engage in light physical activity (an average of 4.62 days a week, about 57.38 min a day). There were significant differences in exercise motivation among youth baseball enthusiasts of different genders in terms of psychology (*t* = 3.558, *p* = 0.018) and fun (*t* = 6.401, *p* < 0.001), as well as significant differences in exercise behavior (*t* = 1.534, *p* = 0.008), the above significant differences in motivation and behavior were greater among males than females, which means that male youth baseball enthusiasts were more affected by psychological factors and fun factors when participating in exercise (Table 2).

**Table 2:** Relationship between motivations and behaviors of exercise participation among youth baseball enthusiasts of different genders.

Factor		Male (*N* = 484)	Female (*N* = 435)	*t*	Cohen’s *d*
		Mean ± SD	Mean ± SD		
Exercise motivation	Competition	3.64 ± 0.97	3.43 ± 0.95	3.421	0.22
	Appearance	3.70 ± 0.92	3.68 ± 0.87	0.426	0.02
	Life	3.33 ± 1.18	3.21 ± 1.09	1.527	0.11
	Social interaction	3.81 ± 0.94	3.53 ± 0.92	4.483	0.30
	Healthy	4.11 ± 0.86	3.96 ± 0.91	2.459	0.17
	Psychology	4.04 ± 0.87	3.82 ± 0.97	3.558^*^	0.24
	Ability	3.72 ± 0.90	3.49 ± 0.97	3.744	0.25
	Fun	4.10 ± 0.90	3.68 ± 1.07	6.401^**^	0.42
Exercise behavior		39.67 ± 30.91	32.96 ± 30.57	1.534^**^	0.22

^*^p < 0.05 and ^**^p < 0.01.

There was a significant positive correlation between youth baseball enthusiasts’ exercise motivation in terms of life, psychology, fun and exercise behavior (Table 3).

**Table 3:** Correlation analysis between exercise participation motivation and behavior (*r* value/power value).

Variables	Competition	Appearance	Life	Social interaction	Healthy	Psychology	Ability	Fun
Exercise behavior	−0.025/0.12	−0.013/0.07	0.545^**^/1	0.109/0.91	0.128/0.97	0.429^**^/1	0.042/0.25	0.439^**^/1
Competition	—	0.648^**^/1	0.262^**^/1	0.534^**^/1	0.524^**^/1	0.416^**^/1	0.643^**^/1	0.387^**^/1
Appearance		—	0.188^**^/0.99	0.473^**^/1	0.538^**^/1	0.403^**^/1	0.569^**^/1	0.342^**^/1
Life			—	0.293^**^/1	0.189^**^/0.99	0.490^**^/1	0.259^**^/1	0.443^**^/1
Social interaction				—	0.596^**^/1	0.493^**^/1	0.599^**^/1	0.492^**^/1
Healthy					—	0.579^**^/1	0.614^**^/1	0.510^**^/1
Psychology						—	0.527^**^/1	0.773^**^/1
Ability							—	0.540^**^/1
Fun								—

^**^p < 0.01.

From the stepwise multiple regression analysis in Table 4, it can be found that the multiple correlation coefficient of the two variables of life and fun was 0.642. The final regression model explained 41.0% of the variance in exercise behavior. This indicates that motivational factors alone account for a substantial proportion of adolescents’ exercise behavior, suggesting meaningful practical relevance for sport promotion and educational interventions. VIF values are below the chosen threshold (VIF <2), demonstrating the absence of problematic multicollinearity.

**Table 4:** Regression analysis of exercise participation motivation and behavior.

Predictor variables	*R*	*R* ^2^	*B*	Beta (β)	Sig.	VIF	Power value
(Constant)			−31.543				1
Life	0.591	0.349	11.815	0.436	<0.001	1.244	
Fun	0.619	0.383	7.524	0.246	<0.001	1.244	

The Equation 1 of the model is as follows:


Exercise behavior=−31.543+11.815×Life+7.524×Fun (1)

## 5 Discussion

This study examined the relationships between motivation and exercise behavior among youth baseball enthusiasts, with a particular focus on gender differences. The results revealed that male participants reported higher psychological motivation, enjoyment motivation, and exercise behavior than females, consistent with previous research indicating greater male engagement in competitive sports contexts (20, 21). As a team sport emphasizing performance and cooperation, baseball may more readily enhance male adolescents’ achievement-related motivation.

Exercise behavior was positively associated with life motivation and enjoyment motivation, supporting self-determination theory (2). When adolescents perceive baseball as enjoyable and meaningful in daily life, intrinsic motivation is strengthened, which may facilitate more sustained participation (22, 23). These findings suggest that enjoyment and lifestyle integration are central motivational drivers of youth sport behavior (24–28).

From a practical perspective, youth sport programs should prioritize autonomy-supportive, enjoyable, and socially engaging training environments to enhance intrinsic motivation and promote long-term participation. Special attention should be given to creating inclusive conditions that support female adolescents’ engagement in baseball (29–36).

Several limitations should be acknowledged. The use of stepwise regression represents an exploratory approach and may be sensitive to sample characteristics, and the cross-sectional design precludes causal inference. Future research should employ theory-driven analytical methods and longitudinal designs and to consider using item reduction techniques or confirmatory factor analysis to further examine the discriminative power of constructs to further clarify motivational mechanisms underlying youth sport participation. Future research could further explore the impact of different age groups or sports experiences on motivation and behavior, or incorporate variables such as social support, family factors, and coaching leadership style to gain a more comprehensive understanding of the dynamics of adolescent sports participation (37–39).

Overall, this study highlights the importance of fun-oriented and lifestyle-based motivational strategies in promoting sustained baseball participation among youth. As the sample was drawn from a specific cultural context, caution is warranted when generalizing these findings to other populations.

## 6 Conclusion

Research results show significant differences in psychological motivation, enjoyment motivation, and exercise behavior among baseball enthusiasts of different genders, with men exhibiting higher overall motivation levels than women. Future baseball promotions should incorporate gender-specific design, such as incorporating more social interaction and fun elements for women to increase their willingness to participate. Furthermore, since exercise behavior is significantly correlated with life motivation, psychological motivation, and enjoyment motivation, coaches and promoters are encouraged to prioritize enhancing students’ life goals and enjoyment of exercise, establishing positive feedback mechanisms, and fostering long-term participation and the development of exercise habits.

## Data Availability

The original contributions presented in the study are included in the article/supplementary material, further inquiries can be directed to the corresponding authors.
